# Patterns and prognosis of regional recurrence in nasopharyngeal carcinoma after intensity‐modulated radiotherapy

**DOI:** 10.1002/cam4.5020

**Published:** 2022-07-13

**Authors:** Xiao‐Tang Xiao, Yi‐Shan Wu, Yu‐Pei Chen, Xu Liu, Rui Guo, Ling‐Long Tang, Jun Ma, Wen‐Fei Li

**Affiliations:** ^1^ Department of Radiation Oncology Sun Yat‐sen University Cancer Centre, State Key Laboratory of Oncology in South China, Collaborative Innovation Centre for Cancer Medicine Guangzhou Guangdong People's Republic of China; ^2^ Department of Nasopharyngeal Carcinoma Sun Yat‐sen University Cancer Center Guangzhou People's Republic of China

**Keywords:** failure pattern, IMRT, nasopharyngeal carcinoma, prognosis, regional recurrence

## Abstract

**Objective:**

We analyzed the patterns of lymph node (LN) failure and prognosis in patients with regional recurrent nasopharyngeal carcinoma (rNPC) alone after primary intensity‐modulated radiotherapy (IMRT).

**Methods:**

A total of 175 patients who were treated with IMRT between 2010 and 2015 and who experienced regional recurrence alone were included. Recurrent LNs were re‐located in the initial pretreatment imaging and IMRT plan and failures were classified as in‐field or out‐field based on target volume delineation. All patients underwent curative salvage treatment. Independent prognostic factors for overall survival (OS) were selected by multivariate Cox analysis.

**Results:**

Level IIb (49.1%, 86/175) was the most frequent recurrence site, followed by level IIa (36%), level III (18.9%), level IVa (12%), the retropharyngeal region (8%), level Va (6.9%), and the parotid region (6.9%). A total of 264 recurrent LNs were recorded: 149 (56.4%) were classified as in‐field failure with a prescribed dose ≥66 Gy, 60 (22.7%) with 60 to <66 Gy, 32 (12.1%) with 50 to <60 Gy, and 23 (8.7%) as an out‐field failure, which mainly occurred in the parotid region and level Ib. After a median follow‐up of 52.8 months, the estimated 5‐year OS rate was 66.9%. Multivariate analysis showed that age, plasma Epstein–Barr virus DNA level, extranodal extension, lower neck involvement, and parotid LN recurrence were independent prognostic factors of OS.

**Conclusions:**

In‐field failure represented the main pattern of regional recurrence and out‐field failure mainly occurred in the parotid gland and level Ib. Patients with regional rNPC alone had a good prognosis after salvage treatment.

## Graphical abstract

Level IIb was the most frequent recurrence site in patients with regional rNPC after primary IMRT. In‐field failure represented the main pattern of regional recurrence and out‐field failure mainly occurred in the parotid gland and level Ib.

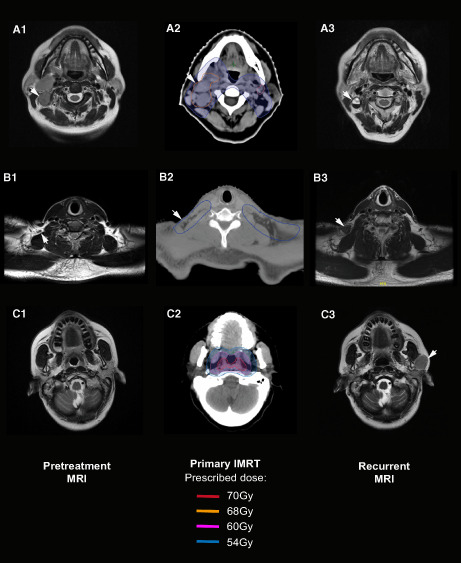

## 1 INTRODUCTION

Nasopharyngeal carcinoma (NPC) is a unique head and neck cancer with an unbalanced endemic distribution. Radiotherapy (RT) is the primary treatment modality for early‐stage NPC, and RT plus chemotherapy is the standard treatment for locoregionally advanced disease.^1^ Currently, intensity‐modulated RT (IMRT) has replaced conventional two‐dimensional RT (2DRT) as the main RT technique for NPC. In patients with NPC, IMRT significantly improves locoregional control and overall survival (OS) and decreases the incidence of radiation‐induced late toxicities as compared with 2DRT.^2^

After IMRT‐based comprehensive treatment, approximately 10% and 5% of patients will experience local recurrence and regional recurrence, respectively.^3^, ^4^, ^5^ Due to the low regional recurrence rate, there remains a lack of large‐scale studies of the regional failure pattern in patients with NPC after primary IMRT. Small‐sample studies have shown that the main pattern of regional recurrence is in‐field failures, with level II being the most common recurrence site.^6^, ^7^ Moreover, because of the rarity of this disease, few studies have reported the treatment outcome and prognostic factors of patients with regional recurrent NPC (rNPC) undergoing salvage surgery and/or reirradiation. Unlike patients with newly diagnosed NPC, patients with regional recurrence are mainly treated with surgery, while only those with unresectable recurrent lymph nodes (LNs) will undergo reirradiation. It is unknown whether the prognostic value of different nodal variables such as LN level, laterality, size, extranodal extension, and necrosis in patients with rNPC differs from those in patients with newly diagnosed NPC.^8^, ^9^, ^10^

Accordingly, we conducted this retrospective study to explore the regional failure patterns of IMRT‐treated patients with NPC, which may provide information on neck node level delineation and dose selection. We also investigated the outcomes and prognostic factors in a relatively large group of patients with regional rNPC following salvage treatment to better predict prognosis and guide individual treatment for patients with rNPC.

## 2 METHODS

### 2.1 Patients

The medical records of patients with newly diagnosed, non‐distant metastatic, histologically proven NPC treated with IMRT at Sun Yat‐sen University Cancer Center between 2010 and 2015 were retrospectively analyzed. Patients who met the following criteria were enrolled in the study: (1) regional recurrence occurred over 6 months after the end of primary IMRT and before June 30, 2020; (2) complete initial pretreatment imaging and recurrent imaging of the head and neck region, including magnetic resonance imaging (MRI), computed tomography (CT) and/or 18F‐fluorodeoxyglucose (18F‐FDG) positron emission tomography CT (PET‐CT), were available; (3) no previous or synchronous local recurrence or distant metastasis; (4) salvage treatments received after recurrence including neck dissection and/or reirradiation (≥60 Gy). Patients with synchronous malignancies or severe coexisting diseases were excluded. The Sun Yat‐sen University Cancer Center institutional review board approved this study.

### 2.2 Initial treatment before recurrence

All patients underwent radical IMRT using the simultaneous integrated boost technique. Tumor target volumes were delineated according to our institutional treatment protocol.^11^ The prescribed doses to the primary gross tumor volume (GTVp) planning target volume (PTV), involved LNs (GTVnd), high‐risk clinical target volume (CTV1), and low‐risk clinical target volume (CTV2) was 66–72, 64–70, 60–63, and 54–56 Gy, respectively, in 28–33 fractions. All patients were treated with one fraction daily over 5 days per week. During the study, the institutional guidelines recommended only IMRT for stage I disease and concurrent chemoradiotherapy (CCRT) with or without induction/adjuvant chemotherapy for stage II–IVA disease.

### 2.3 Diagnosis of regional recurrence

Regional recurrence was diagnosed by histology and PET‐CT, MRI, CT, and/or sonography. Plasma Epstein–Barr virus (EBV) DNA concentrations were routinely measured by qPCR.^12^ To increase diagnostic accuracy, two experienced doctors retrospectively confirmed all regional recurrence diagnoses based on histology, abnormal imaging findings, and/or progressive disease. Recurrent tumors were classified according to the 8th edition of the Union for International Cancer Control and American Joint Committee on Cancer (UICC/AJCC) staging system.^13^ In the present study, parotid LN recurrence was classified as a rN3 disease.^14^ LN location was based on the international consensus guidelines for neck node level delineation^15^ and only the largest recurrent LN at each level was recorded. The maximal axial diameter (MAD), minimal axial diameter (MIAD), laterality, extranodal extension, and necrosis of the recurrent LNs were measured and recorded. Lower neck involvement referred to level IV or Vb invasion below the caudal edge of the cricoid cartilage.^16^ The criteria for diagnosing extranodal extension on MRI were the presence of indistinct nodal margins, irregular nodal capsular enhancement, or infiltration into the adjacent fat or muscle.^17^ The diagnostic criteria for necrosis included a high‐signal intensity focal area on T2‐weighted images or a low‐signal intensity focal area on contrast‐enhanced T1‐weighted images.^18^ The recurrent LNs were re‐located at the same sites in the initial diagnostic imaging and primary IMRT plan. The failure patterns were defined as follows: recurrent LNs included in the CTV2 were defined as in‐field failure while those outside the CTV2 were defined as out‐field failure.

### 2.4 Treatment after regional recurrence

The salvage regional treatments included neck dissection and reirradiation alone or in combination. The choice of salvage treatment was determined by the tumor size, extent, and location, and the patient's intentions and consultation with radiation oncologists and surgeons. The summarized treatment modalities are as follows: (1) Either radical or selective neck dissection was performed for recurrent neck disease; (2) Reirradiation with IMRT was suitable for irresectable LNs but the interval between the first and second courses of IMRT should be ≥1 year. The gross recurrent LNs (rGTVnd) and the CTV included the entire LN‐positive regions. The prescribed doses were 60–70 Gy to the rGTVnd and 50–54 Gy to the CTV in 27–35 fractions,^19^ (3) in selected patients, chemotherapy and targeted therapy were used as multidisciplinary treatment combined with surgery or reirradiation.

### 2.5 Statistical analysis

Patients were assessed every 3 months during the first 3 years and every 6 months thereafter until death. The recurrence interval was measured from the end of primary IMRT to the day of locoregional recurrence. The primary endpoint was OS, which was calculated from the day of regional recurrence to the date of last follow‐up or death. The LN level involvement in patients with primary and recurrent NPC was compared using the McNemar test. Survival rates were calculated using the Kaplan–Meier method and survival differences were compared by the log‐rank test. Multivariate analyses with the Cox regression model^20^ were carried out to calculate hazard ratios (HRs) and 95% confidence intervals (CIs) and to identify significant independent prognostic factors by backward elimination. The statistical analyses were performed using SPSS 26. Two‐tailed *p*‐values <0.05 were considered statistically significant.

## 3 RESULTS

### 3.1 Patient characteristics

**FIGURE 1:**
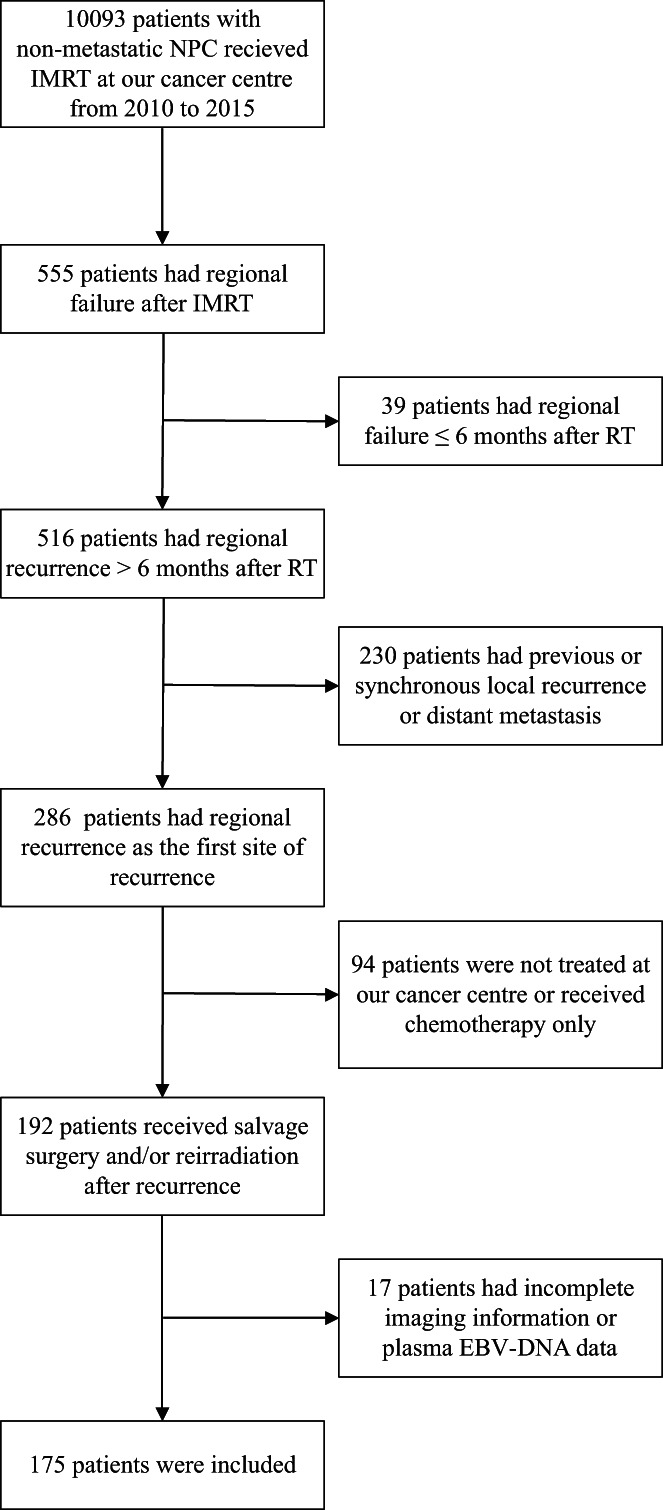
Flow diagram of the study selection process (inclusion and exclusion criteria)

In total, 175 eligible patients with regional recurrence alone were included in this study (Figure 1). Table 1 shows the patients' characteristics. There were more male patients than female patients (3.6:1 ratio) and the median age was 47 years (range, 22–77 years). Up to 99.4% (174/175) of the recurrences were diagnosed by pathology and 64.6% of the patients (113/175) had detectable plasma EBV DNA at recurrence. The median recurrence time since primary IMRT completion was 18.6 months (range, 6.2–85 months). Overall, 136 patients (77.7%) were classified as rN1, 6 (3.4%) as rN2, and 33 (18.9%) as rN3.

**TABLE 1:** Patient characteristics

Characteristic	Number of patients (%) (*N* = 175)
Sex	
Male	137 (78.3%)
Female	38 (21.7%)
Age at recurrence	
<60 years	157 (89.7%)
≥60 years	18 (10.3%)
Method for diagnosis of recurrence	
Pathology	174 (99.4%)
PET‐CT/MRI	1 (0.6%)
EBV DNA level at recurrence	
0 copy/mL	62 (35.4%)
>0–<4000 copies/mL	71 (40.6%)
≥4000 copies/mL	42 (24%)
Recurrent *N* stage	
rN1	136 (77.7%)
rN2	6 (3.4%)
rN3	33 (18.9%)
No. of recurrent lymph nodes	
Solitary	103 (58.9%)
Multiple	72 (41.1%)
MAD of recurrent lymph nodes	
<20 mm	146 (83.4%)
≥20 mm	29 (16.6%)
Laterality	
Unilateral	166 (94.9%)
Bilateral	9 (5.1%)
Lower neck involvement^a^	
No	153 (87.4%)
Yes	22 (12.6%)
Extranodal extension	
No	111 (63.4%)
Yes	64 (36.6%)
Necrosis	
No	130 (74.3%)
Yes	45 (25.7%)
Regional treatment after recurrence	
Surgery	148 (84.6%)
Reirradiation	15 (8.6%)
Surgery + reirradiation	12 (6.9%)
Chemotherapy	
No	143 (81.7%)
Yes	32 (18.3%)

Abbreviations: EBV, Epstein–Barr virus; IMRT, intensity‐modulated radiotherapy; MRI, magnetic resonance imaging; PET, positron emission tomography; CT, computed tomography; MAD, maximal axial diameter.

^a^ Cervical lymph nodes below the caudal border of the cricoid cartilage.

### 3.2 Patterns of regional recurrence

Of the 175 patients, 103 (58.9%) experienced solitary nodal recurrence and 72 (41.1%) had multiple nodal recurrences; 166 patients (94.9%) had unilateral recurrence while only 9 (5.1%) had a bilateral recurrence. Overall, 29 patients (16.6%) had recurrent LNs ≥20 mm, 64 (36.6%) had extranodal extension, and 45 (25.7%) had nodal necrosis. Table 2 shows the comparisons of the distributions of recurrent and initial LNs. Level IIb (49.1%, 86/175) was the most frequent recurrence site, followed by level IIa (36%, 63/175), level III (18.9%, 33/175), level IVa (12%, 21/175), the retropharyngeal region (8%, 14/175), level Va (6.9%, 12/175), and the parotid region (6.9%, 12/175).

**TABLE 2:** Comparison of lymph node level involvement in 175 patients with regional rNPC and pNPC

Lymph node level	Number of patients (%)		*p*
	Involvement in regional rNPC	Involvement in pNPC	
RP region	14 (8%)	155 (88.6%)	<0.001
Level IIa	63 (36%)	164 (93.7%)	0.008
Level IIb	86 (49.1%)	168 (96%)	<0.001
Level III	33 (18.9%)	126 (72%)	<0.001
Level IVa	21 (12%)	25 (14.3%)	0.597
Level Va	12 (6.9%)	33 (18.9%)	0.001
Level Vb	6 (3.4%)	11 (6.3%)	0.327
Level Ia	2 (1.1%)	1 (0.6%)	1.000
Level Ib	8 (4.6%)	5 (2.9%)	1.000
Parotid region	12 (6.9%)	2 (1.1%)	0.133
Level Xa	1 (0.6%)	0 (0%)	‐

Abbreviations: rNPC, recurrent nasopharyngeal carcinoma; pNPC, primary nasopharyngeal carcinoma; RP, retropharyngeal.

### 3.3 Dosimetric analysis of primary IMRT

**FIGURE 2:**
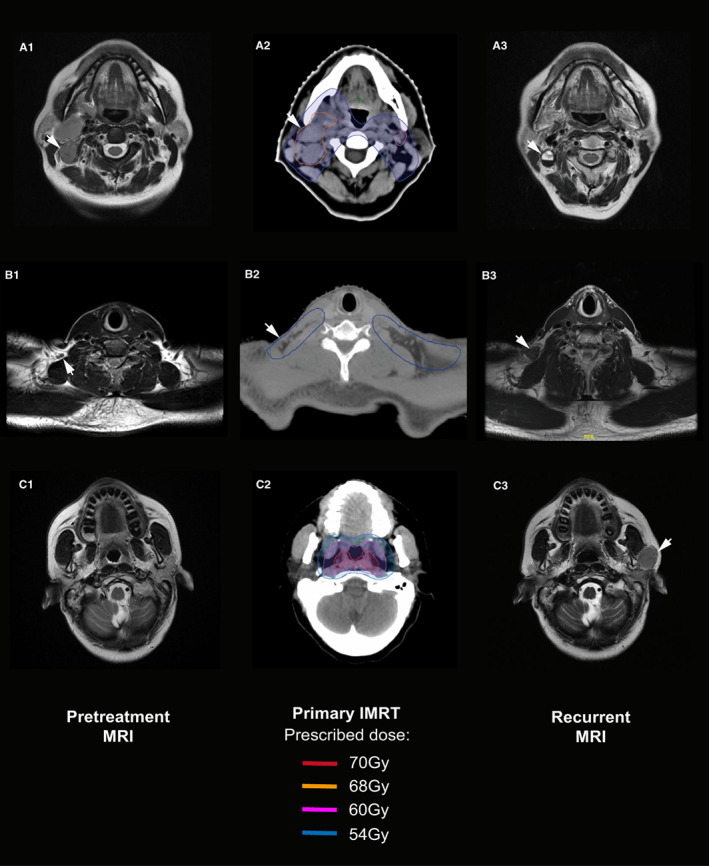
Three patients with solitary lymph node (LN) recurrence: in‐field failure at level IIb with a prescribed dose of 68 Gy (A1–A3); in‐field failure at level Va with a prescribed dose of 54 Gy (B1–B3), and out‐field failure in the parotid region (C1–C3). Left: Initial diagnostic magnetic resonance imaging (MRI). Middle: Target volume delineation and dose prescription of primary intensity‐modulated radiotherapy. Right: MRI at recurrence. Arrows indicate the recurrent LNs and pretreatment LNs re‐located at the same site on the diagnostic MRI.

Only the largest recurrent LN in each region was recorded. A total of 264 recurrent LNs in the 175 patients were recorded and re‐located in the initial diagnostic imaging and IMRT plan. In total, 155 patients (88.6%) had in‐field failure alone, 13 (7.4%) had an out‐field failure alone, and 7 (4%) experienced both in‐field and out‐field failure. Of the 264 recurrent LNs, 152 patients (57.6%) had initial LNs with MIAD ≥10 mm, 103 (39%) had LNs with MIAD <10 mm, and 9 (3.4%) had no obvious LNs at the same sites on initial diagnostic imaging. Table 3 shows the patterns of the 264 recurrent LNs and the evaluation of the initial LN dose. The median prescribed dose was 70 Gy to the initial LNs in the retropharyngeal region, 66 Gy to levels II–IV, and 56 Gy to level V. Overall, 149 nodal failures (56.4%) were classified as in‐field failure with a prescribed dose ≥66 Gy, 60 (22.7%) with a prescribed dose of 60 Gy to <66 Gy, 32 (12.1%) with a prescribed dose of 50 Gy to <60 Gy, and 23 (8.7%) were classified as out‐field failures. Of the 149 in‐field failures with a prescribed dose ≥66 Gy, 133 (89.3%) occurred at levels II, III, and in the retropharyngeal region. Of the 60 in‐field failures with a prescribed dose of 60 Gy to <66 Gy, 56 (93.3%) occurred at levels II and III. Of the 32 in‐field failures with a prescribed dose of 50 Gy to <60 Gy, 19 (59.4%) occurred at levels IV and V. Of the 23 out‐field failures, 12 (52.2%) occurred in the parotid region and 7 (30.4%) at level Ib, where only small or no obvious LNs were detected on initial diagnostic imaging (Figure 2). Only one patient had a parotid LN with MIAD ≥10 mm before initial treatment and experienced out‐field failure due to physician negligence.

**TABLE 3:** Patterns of the 264 recurrent lymph nodes and evaluation of initial lymph node dose

LN level	No. of recurrent LNs (solitary recurrence)	No. of in‐field failures with a prescribed dose of ≥66 Gy		No. of in‐field failures with a prescribed dose of 60 to <66 Gy		No. of in‐field failures with a prescribed dose of 50 to <60 Gy			No. of out‐field failures		
		Initial LNs with MIAD ≥10 mm	Initial LNs with MIAD <10 mm	Initial LNs with MIAD ≥10 mm	Initial LNs with MIAD < 10 mm	Initial LNs with MIAD ≥10 mm	Initial LNs with MIAD <10 mm	No initial LNs	Initial LNs with MIAD ≥10 mm	Initial LNs with MIAD <10 mm	No initial LNs
RP region	14 (9)	8	4	0	1	1	0	0	0	0	0
Level IIa	65 (32)	34	8	16	4	2	1	0	0	0	0
Level IIb	89 (41)	47	8	23	6	2	3	0	0	0	0
Level III	34 (3)	11	13	3	4	0	3	0	0	0	0
Level IVa	21 (4)	2	9	0	2	1	6	0	0	1	0
Level Va	12 (3)	0	4	0	0	0	7	1	0	0	0
Level Vb	6 (0)	0	1	1	0	0	4	0	0	0	0
Level Ia	2 (0)	0	0	0	0	0	0	0	0	2	0
Level Ib	8 (3)	0	0	0	0	0	1	0	0	6	1
Parotid	12 (8)	0	0	0	0	0	0	0	1	5	6
Level Xa	1 (0)	0	0	0	0	0	0	0	0	0	1
Total	264 (103)	102	47	43	17	6	25	1	1	14	8

Abbreviation: LN, lymph node; MIAD, minimal axial diameter; RP, retropharyngeal.

### 3.4 Treatment outcome after recurrence

**FIGURE 3:**
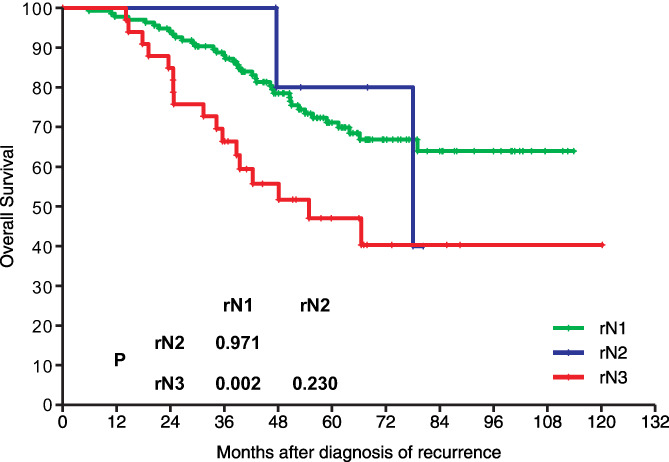
Kaplan–Meier overall survival curves for different recurrent N stages

After recurrence, all patients underwent salvage reirradiation and/or surgery: 148 patients (84.6%) underwent surgery alone, 15 (8.6%) underwent reirradiation alone, and 12 (6.9%) underwent surgery plus reirradiation. In patients receiving reirradiation alone, the median dose was 64 Gy (range, 60–70 Gy). Overall, 32 patients (18.3%) underwent chemotherapy combined with locoregional treatment. Adjuvant chemotherapy was delivered to 15 patients, induction chemotherapy to 7 patients, CCRT to 7 patients, and induction chemotherapy plus CCRT to 3 patients. After a median follow‐up of 52.8 months (range, 5.9–120.2 months), the estimated 5‐year OS rate was 66.9%. In total, 57 patients (32.6%) died: 51 (89.5%) from tumor progression, 1 (1.8%) from pneumonia, 2 (3.5%) from poor nutrient status caused by feeding difficulty, and 3 (5.3%) from unknown reasons. The estimated 5‐year OS rate was 71.1% for rN1, 80% for rN2, and 47% for rN3 (Figure 3).

### 3.5 Univariate and multivariate analyses

To determine the prognostic factors associated with OS, the univariate and multivariate analyses included the patient's sex, age, plasma EBV DNA level at recurrence, number of recurrent LNs, laterality, lower neck involvement, MAD, nodal necrosis, extranodal extension, parotid LN involvement, and treatment after recurrence. Only age, plasma EBV DNA level, lower neck involvement, extranodal extension, and parotid LN recurrence were independent prognostic factors for OS in the multivariate analyses (Table 4).

**TABLE 4:** Univariate and multivariate analyses of prognostic factors for overall survival

Variable	Univariate analysis			Multivariate analysis		
	HR	95% CI	*p*	HR	95% CI	*p*
**Sex** female vs. male	0.70	0.35–1.38	0.304	0.68	0.32–1.43	0.307
**Age at recurrence** ≥ 60 vs. < 60 years	1.56	0.74–3.30	0.243	2.34	1.08–5.09	0.031
**EBV DNA level**			<0.001			<0.001
>0–<4000 vs. 0 copies/mL	0.96	0.48–1.91	0.900	0.76	0.37–1.54	0.441
≥4000 vs. 0 copies/mL	3.24	1.73–6.08	<0.001	2.91	1.47–5.77	0.002
**No. of recurrent nodes** multiple vs. solitary	1.92	1.14–3.23	0.014	1.26	0.68–2.35	0.466
**Laterality** bilateral vs. unilateral	0.52	0.13–2.14	0.365	0.55	0.13–2.33	0.414
**Lower neck involvement** yes vs. no	2.26	1.17–4.38	0.015	2.07	1.03–4.16	0.041
**MAD of recurrent LNs** ≥ 20 vs. < 20 mm	2.24	1.22–4.10	0.009	1.34	0.65–2.75	0.426
**Necrosis** yes vs. no	1.00	0.55–1.80	0.989	1.78	0.92–3.45	0.088
**Extranodal extension** yes vs. no	1.79	1.06–3.02	0.029	1.79	1.00–3.18	0.049
**Parotid LN recurrence** yes vs. no	2.36	1.07–5.22	0.034	3.65	1.59–8.37	0.002
**Reirradiation** yes vs. no	1.24	0.61–2.53	0.555	1.05	0.48–2.33	0.903
**Chemotherapy** yes vs. no	1.85	1.01–3.38	0.048	1.37	0.68–2.78	0.376

Abbreviations: HR, hazard ratio; CI, confidence interval; IMRT, intensity‐modulated radiotherapy; EBV, Epstein–Barr virus; MAD, maximal axial diameter.

## 4 DISCUSSION

Our study showed that level II was the most frequent site of regional recurrence and that in‐field failure was the main recurrence pattern after IMRT in patients with NPC, which is concurrent with previous studies.^6^, ^7^, ^21^ Xue et al. analyzed 17 patients with regional rNPC after IMRT, of which 94.1% (16/17) had in‐field failure. They found that recurrence occurred most frequently at level II (70.6%) and the retropharyngeal area (52.9%), followed by levels III and IV, which suggested that the jugular LN chains had a similar frequency of relapse to the distribution patterns of metastatic LNs at diagnosis.^7^ However, we determined that the recurrence rate of retropharyngeal LNs was only 8%, which was lower than the 52.9% reported by Xue et al.^7^ This is probably because, at our center, the retropharyngeal LNs were delineated in the GTVp and irradiated with 70 Gy, while the median prescribed dose for level II–IV recurrent LNs was only 66 Gy. A higher radiation dose may contribute to the lower recurrence rate in the retropharyngeal region. The majority of in‐field failures with a prescribed dose of 50 Gy to <60 Gy occurred at levels IV–V with initial MIAD <10 mm. Therefore, the diagnosis of level IV–V LNs with MIAD <10 mm should be confirmed before the initial treatment, especially for patients with enlarged LNs at levels II–III. The radiation dose should be standardized according to evidence‐based guidelines, and positive LNs must be given a radical dose.

As parotid LN metastasis is seldom observed in newly diagnosed NPC (<3%),^14^, ^22^, ^23^ the parotid region is not routinely prophylactically irradiated unless there is a positive LN. However, approximately 5.9–40% of nodal failures occurred in the parotid region in patients with NPC treated with IMRT.^6^, ^7^ Cao et al. reported 10 NPC cases with parotid LN recurrence after IMRT: 3 were in‐field failures, 7 were marginal failures, and none were out‐field failures. To reduce the risk of parotid LN recurrence, they suggested reducing the dose constraint criteria to the ipsilateral parotid gland, especially for patients with lateral retropharyngeal lymphadenopathy.^24^ Li et al. reported that parotid LN recurrence accounted for all out‐field failures, and most cases had sub‐centimeter, nonspecific nodules at the same site in the parotid gland on the pretreatment MRI despite a negative PET‐CT scan.^6^ In our study, parotid LN recurrence accounted for 6.9% of all nodal failures, all of which were out‐field failures. Moreover, 41.7% (5/12) of patients with parotid LN recurrence had sub‐centimeter lesions at the same site on initial diagnostic imaging. Therefore, we recommend a comprehensive assessment that includes imaging, fine‐needle aspiration, or biopsy for suspicious nodules in the parotid region before treatment, especially for patients with multilevel nodal disease, to minimize the chance of dose omission.

Given the low incidence of level Ib LN involvement (approximately 3%),^22^, ^23^ elective irradiation of level Ib LNs is recommended for patients with NPC.^25^ Guo et al. reported that 22 patients with NPC experienced regional recurrence after level Ib‐sparing IMRT and 4 patients (18.2%) had an out‐field failure at level Ib.^26^ However, other studies have reported that no patient had level Ib recurrence after the omission of level Ib irradiation.^27^, ^28^, ^29^ In the present study, 4.6% of the patients had level Ib recurrence, most of which were out‐field failures with sub‐centimeter nodules at the same sites on initial diagnostic imaging. Moreover, most patients with level Ib recurrence had at least one of the following high‐risk features on initial diagnostic imaging: involvement of level II LNs with extranodal extension, level II nodal involvement with MAD >2 cm, and involvement of the anterior half of the nasal cavity.^25^ Therefore, a comprehensive pretreatment assessment is also recommended for level Ib LNs, and prophylactic coverage of ipsilateral level Ib LNs should be performed in high‐risk patients according to the international guidelines for delineating the CTV for NPC.^25^

The survival and prognostic factors of patients with NPC with regional recurrence alone have seldom been reported. A retrospective study of 348 patients with regional rNPC treated with neck dissection reported that >70% of patients were classified as rN1 stage, and the 3‐year OS rate was 79.3%. Moreover, multivariate analysis revealed that microscopic positive LN >2, extranodal extension, and lower neck involvement correlated negatively with OS.^9^ Yeung et al. reported that an absolute number of positive LNs >5 and LN density > 20% were significantly associated with poorer OS, while extranodal extension and pathological N stage did not affect OS following a neck dissection for regional residual or recurrent NPC.^10^ You et al. reported that patients with resectable disease had significantly better OS and classified rN1‐resectable, rN2‐resectable, and rN3‐resectable as rN1 in the proposed surgical tumor‐node‐metastasis (TNM) stage.^8^ In the present study, most patients had rN1 disease and underwent salvage neck dissection. The estimated 5‐year OS rate was 66.9%. In multivariate analysis, age ≥ 60 years, high plasma EBV DNA level, extranodal extension, lower neck involvement, and parotid LN recurrence were negatively associated with OS, and have also been identified as adverse prognostic factors in primary NPC.^14^, ^16^, ^17^, ^30^ However, necrosis was not a poor prognosticator in the present study, probably because >90% of the patients with recurrent LNs were treated with surgery but not reirradiation.^31^ Our results indicate that patients with regional rNPC with one or more of these negative prognostic factors are candidates for more aggressive therapeutic strategies, such as combined local and systemic therapies.

The present study has several limitations. First, only patients with regional rNPC who underwent neck dissection or salvage RT (≥60 Gy) were included. Therefore, the proportion of early recurrent disease was higher than the actual number. Second, the use of retrospective data introduced the possibility of selection bias. For example, patients undergoing chemotherapy had poorer survival in univariate analysis but not in multivariate analysis, which may largely be explained by the selection bias. Therefore, further investigation is warranted to identify patients with rNPC who may benefit from systemic chemotherapy.

## 5 CONCLUSION

Our study shows that in‐field failure is the main pattern of regional failure in patients with NPC treated with IMRT, and level II is the most common recurrence site. Out‐field failure mainly occurs in the parotid gland and level Ib. More attention should be paid to the small LNs in levels IV, V, Ib, and the parotid gland. Curative salvage neck dissection and/or reirradiation (≥60 Gy) achieved excellent long‐term survival in patients with regional rNPC alone.

### AUTHOR CONTRIBUTION

X.T.X., Y.S.W., and W.F.L. contributed to the study design and conception. X.T.X., W.F.L., X.L., Y.P.C., R.G., and L.L.T. contributed to data acquisition. X.T.X., Y.S.W., and W.F.L. analyzed and interpreted the data. X.T.X. and W.F.L. contributed to manuscript preparation and editing. J.M. and W.F.L. contributed to quality control and review of the data and manuscript. All authors have read and approved the final version of the submitted manuscript.

## CONFLICT OF INTEREST

The authors declare that no competing interests exist.

## ETHICS STATEMENT

The study was approved by the institutional review board of Sun Yat‐sen University Cancer Center (No. B2021‐330‐01) and performed according to the institutional policy for protecting patients' confidential information. The need for informed consent was waived.

## Data Availability

Data available on request from the authors.
